# Comparative Evaluation of Antimicrobial, Antiamoebic, and Antiviral Efficacy of Ophthalmic Formulations

**DOI:** 10.3390/microorganisms10061156

**Published:** 2022-06-04

**Authors:** Ciro Caruso, Daniela Eletto, Alessandra Tosco, Martina Pannetta, Fabio Scarinci, Mario Troisi, Amalia Porta

**Affiliations:** 1 Corneal Transplant Centre, Pellegrini Hospital, 80134 Naples, Italy; cirocarusoeye@gmail.com; 2 Department of Pharmacy, University of Salerno, 84084 Fisciano, Italy; tosco@unisa.it (A.T.); mpannetta@unisa.it (M.P.); 3 Department of Ophthalmology, IRCCS Fondazione, Bietti, Via Livenza, 3, 00198 Roma, Italy; fabioscarinci@gmail.com; 4 Department of Neurosciences, Reproductive Sciences and Dentistry, Eye Clinic, University of Naples “Federico II”, 80138 Naples, Italy; troisi165@gmail.com

**Keywords:** antiseptics, antibacterial, antiamoebic, antiviral, ophthalmic solution, eye drops, chlorhexidine, thymol

## Abstract

The extensive use of ophthalmic antibiotics is contributing to the appearance of resistant bacterial strains, which require prolonged and massive treatments with consequent detrimental outcomes and adverse effects. In addition to these issues, antibiotics are not effective against parasites and viruses. In this context, antiseptics could be valuable alternatives. They have nonselective mechanisms of action preventing bacterial resistance and a broad spectrum of action and are also effective against parasites and viruses. Here, we compare the in vitro antibacterial, antiameobic, and antiviral activities of six ophthalmic formulations containing antiseptics such as povidone-iodine, chlorhexidine, and thymol against Gram-positive and Gram-negative bacteria, the amoeba *Acanthamoeba castellanii*, and two respiratory viruses, HAdV-2 and HCoV-OC43. The results suggest that, among all the tested formulations, Dropsept, consisting of Vitamin E TPGS-based (tocopheryl polyethylene glycol succinate) in combination with the antiseptic chlorhexidine, is the one with the highest range of activities, as it works efficiently against bacteria, amoeba, and viruses. On the other hand, the solution containing PVA (polyvinyl alcohol) and thymol showed a promising inhibitory effect on *Pseudomonas aeruginosa*, which causes severe keratitis. Given its high efficiency, Dropsept might represent a valuable alternative to the widely used antibiotics for the treatment of ocular infections. In addition to this commercial eye drop solution, thymol-based solutions might be enrolled for their natural antimicrobial and antiamoebic effect.

## 1. Introduction

Parasitic and bacterial ocular infectious diseases, such as blepharitis, conjunctivitis, keratitis, and endophthalmitis, are responsible for visual morbidity and blindness worldwide [1,2].

Ocular parasitosis is mainly caused by protozoa, helminths, arthropods [3], and, among others, the most common protozoan parasites primarily infecting the ocular tissues are *Acanthamoeba* and *Toxoplasma* gondii species [4,5,6,7,8,9]. These parasites can escape the host’s immune mechanisms by forming dormant cysts within the tissue, thus increasing the risk of chronic ocular infections [3].

Regarding bacterial infections, Gram-positive bacteria account for greater than 70% of the isolated bacterial strains from patients with ocular infections, including *Staphylococcus aureus*, *Staphylococcus epidermidis*, *Streptococcus pneumoniae*, and several other species of *Streptococci* [2]. On the contrary, Gram-negative bacteria are responsible only for 25% of patient-isolated bacterial strains, and the most commonly identified are *Pseudomonas aeruginosa*, *Escherichia coli*, and *Klebsiella pneumonia* [10,11].

In addition, the eye may represent either a potential site of virus replication or an access door for favoring the spreading of the virus to extraocular sites. This phenomenon is particularly concerning if we consider respiratory viruses (including adenovirus, influenza virus, respiratory syncytial virus, coronavirus, and rhinovirus), which can also cause severe diseases in humans, such as acute respiratory failure [12].

Ocular infections are commonly treated with eye drops, topical liniments, and antibiotics gels. To date, some of the most effective antibiotics against eye infections are fluoroquinolones (FQ) and chloramphenicol, followed by tetracyclines, ampicillin, and aminoglycosides [11]. Unfortunately, the use of ophthalmic topical antibiotics with sub-dosed, long-term, and repetitive treatments causes the appearance of resistant bacterial strains in the commensal flora, making the pathogens’ clearance difficult. Moreover, considerable evidence reports clinical failure or unfavorable outcomes when using antibiotics [2,13,14] and also adverse effects and toxicity in some cases. For example, FQs have been reported to cause photosensitization, allergic reactions, and toxicity in specific eye regions [10,15].

In addition to these issues, antibiotics are poorly effective against bacterial biofilms that are often observed in contact lens (CL) users [16,17]. During contact lens (CL) usage, microbial adhesion and bacterial biofilm formation are crucial threats to eye health. Indeed, severe keratitis in humans and animal models is frequently observed when CL surfaces are compromised by bacterial biofilms [18,19]. Although bacteria are not the only pathogens affecting CL wearers, rare but severe keratitis is also caused by parasites, particularly *Acanthamoeba* [20].

The inability of antibiotics to eradicate parasites and biofilms surrounding bacteria, and the increasing risk of antibiotic-resistant species, point out the urgent need for a valid alternative for treating ocular infections and for limiting viral access at the same time. Based on the arguments above, a suitable approach to prevent and solve parasitic and bacterial biofilm-related infections is to use topical treatments that could combine parasite-killing and bactericidal actions along with the ability to control and affect bacterial biofilm and viral replication. In this perspective, antiseptics eye drops could represent a valid alternative to conventional treatments. Indeed, it is known that antiseptics do not trigger the development of bacterial resistance, as they do not have a selective mechanism of action differently from antibiotics [21,22]. In addition, their wide range of mechanisms makes them also suitable as antiviral molecules, as suggested by recent evidence [23]. The most used antiseptics in ophthalmology are povidone-iodine (PVP-I) and chlorhexidine (CHX), which both have microbicidal activity [24]. In particular, CHX is effective against Gram-positive and Gram-negative bacteria, fungi, Chlamydia trachomatis, and *Acanthamoeba* [25,26]. Since CHX has strong antimicrobial activity and relatively low toxicity for mammalian cells, it is considered the most helpful and safest disinfectant. PVP-I is the traditional antiseptic cleansing agent of choice for surgery, with a broad spectrum of activity against bacteria, protozoa, and viruses. Still, it is considered less efficient than CHX in preventing infections post-surgery [27]. In addition to the antiseptics mentioned above, there are many natural compounds of particular interest in medicine because of their microbicidal features and low toxicity and costs. One of the phytochemicals widely used in pharmacology is thymol. Thymol is a monoterpene phenol extracted from Thymus plants, reported as a safe food additive according to the United States Food and Drug Administration [28], and widely used as an antioxidant, food preservative, and flavoring. It is also used in medicine in several fields, as a medical antimicrobial agent, and in applications for wound healing, ulcerative colitis, and rheumatoid arthritis. In ophthalmology, it is now arising as a promising agent in treating eye infections [29].

The present study aimed to compare the antimicrobial, antiamoebic, and antiviral profiles of six ophthalmic formulations containing alternatively antiseptics, commercially available, such as PVP-I, CHX, or natural antiseptics as thymol, not yet in commercial formulations. The comparison of their activities would help to establish the most suitable strategy for counteracting bacterial, parasitic, and, ultimately, viral ocular infections.

## 2. Materials and Methods

### 2.1. Ophthalmic Formulations

The ophthalmic formulations used in this work and their respective composition are listed in Table 1. Thymol (Sigma, T-0501, 99.5%, Steinheim, Germany) was prepared at 500 mg/mL (50% *w*/*v*) in ethanol.

### 2.2. Minimum Inhibitory Concentrations and Minimum Bactericidal Concentrations

The antibacterial activity of each ophthalmic formulation was evaluated against *S. aureus* (ATCC 6538), *S. epidermidis* (ATCC 03111), *E. coli* (ATCC 25922), and *P. aeruginosa* (ATCC 9027). Microorganisms were obtained from LGC Standards S.r.L. (Milan, Italy).

To determine the in vitro minimum inhibitory concentration (MIC) of each solution, micro-broth dilution assays were performed in line with the Clinical and Laboratory Standards Institute (CLSI) guidelines. In detail, two colonies of bacteria from Mueller–Hinton Agar (MHA, Biolife SRL, Milan, Italy) were first individually resuspended at a concentration of ≈1.5 × 10^8^ CFU/mL in sterile saline, then further diluted at a concentration of ≈10^6^ CFU/mL in fresh cation-adjusted Mueller–Hinton Broth (MHB, Biolife SRL, Milan, Italy). From this suspension, 100 μL was used to inoculate flat-bottom 96-well polystyrene microtiter plates containing two-fold serial dilutions from 100 to 6.25 μL/mL of each formulation listed in Table 1.

Two-fold serial dilutions from 100 to 6.25 μL/mL of each formulation listed in Table 1 were placed in 96-well sterile microtiter plates containing Mueller–Hinton Broth (MHB), Biolife SRL, Milan, Italy). Inocula were prepared by diluting overnight cultures (37 °C/18–24 h) at a concentration of ≈10^6^ CFU/mL in fresh MHB. Aliquots of 100 μL were then added to each well, resulting in a final volume of 200 μL and approximately 10^5^ CFU/mL per well. The negative control consisted of 100 μL of MHB and 100 μL of cell suspension.

Based on previously published results, Dropsept was also tested within a narrower range of dilutions from 30 to 6.25 μL/mL (30, 27.5, 25.5, 20, 18, 12.5, 9, 6.25). Microtiter plates were incubated for 24 h at 37 °C. MICs were then determined by reading each bacterial culture in a spectrophotometer set at 600 nm. MIC_50_ and MIC_100_ were calculated as the lowest concentration (µL/mL), causing 50% or 100% growth inhibition. The minimum bactericidal concentration (MBC) was determined by plating 100 μL of each well or proper serial dilutions on MHA incubated at 37 °C for 24 h. The MBC was identified as the lowest concentration that prevents any microbial growth on an agar plate. Each assay was performed in triplicate on separate days.

### 2.3. Disk Diffusion Assay

For the agar disk diffusion assay [30], each microorganism grown overnight in MHB was resuspended at a concentration of ≈2 × 10^8^ CFU/mL in fresh MHB and streaked on MHA plates with a sterile swab. Successively, Whatman filter paper n^o^ 1 was used to prepare disks of approximately 6 mm in diameter, which were sterilized in a hot air oven and then gently pressed onto an agar plate by flame-sterilized forceps and wet with 10 μL of each formulation. After 24 h of incubation at 37 °C, a qualitative analysis of the antimicrobial effect was estimated as an inhibition growth zone. Geneticin was used as a positive and internal control for each bacterial strain, at 1 μg/mL for all bacterial strains and 10 μg/mL for *P. aeruginosa*. Each assay was performed in triplicate on separate days.

### 2.4. Challenge Test

The microbial barrier properties of each formulation were evaluated by the in vitro challenge test described by the European Pharmacopeia to estimate potential contaminations during their use. Each formulation was challenged respectively with the following bacteria: *S. aureus* (ATCC 6538), *S. epidermidis* (ATCC 03111), *E. coli* (ATCC 25922), and *P. aeruginosa* (ATCC 9027). Microorganisms were obtained from LGC Standards S.r.L. (Milan, Italy). According to the standard methodology, 2 mL of each formulation was inoculated with 10^6^/mL bacteria in a flat-bottom 12-well polystyrene plate and incubated at 37 °C. At different time points, 2 h, 24 h, and 7 days, 100 μL aliquots from each well was serially diluted in MHB and plated in duplicate on MHA. Plates were incubated at 37 °C for 24 h, and raw data counts were converted to log_10_ values. Each assay was performed in triplicate on separate days.

### 2.5. Amoebicidal Activity

The amoebicidal activity of each ophthalmic solution was evaluated against *Acanthamoeba castellanii* (ATCC 50370, LGC Standards S.r.L. Milan, Italy) by considering the minimum trophozoite inhibitory concentration (MTIC_50_) as 50% inhibition of *A. castellanii* trophozoites replication compared with controls. To determine the MTIC_50_, serial two-fold dilutions of each formulation were made in Ringer’s solution pH 7.4 (0.125 M NaCl, 5 mM KCl, 1.5 mM CaCl_2_) and incubated with 100 μL of 2 × 10^4^/mL (flat-bottom 96-well plate) axenic trophozoites in growth medium (ATCC Medium: 712 PYG with additives) for about 48 h at 25 °C. At the end of the incubation, six pictures per well were taken by AME-3206 Digital Inverted Microscope (AMG/EVOS, Mill Creek, WA, USA) at 10× magnification, and the amoebas’ ability to proliferate in each tested condition was determined by counting the number of trophozoites [31]. Moreover, a qualitative analysis of the shape of the amoebae and the presence of cell lysis was assessed by observing samples at 40× magnification. Each assay was performed in triplicate on separate days.

### 2.6. Viral Strains and Cell Culture Conditions

Human adenovirus 2 (HAdV-2) (ATCC VR-846) and human coronavirus OC43 (HCoV-OC43) (ATCC VR-1558) were propagated respectively in A549 cells (ATCC CCL-185) or MRC-5 (ATCC CCL-171) (LGC Standard s.r.l., Milan, Italy). Cell incubation, viral propagation, and viral infectivity assessment methods were similar to those suggested by the supplier. Briefly, A549 or MRC-5 cells were cultured at 37 °C with 5% CO_2_ with High Glucose-Dulbecco’s Modified Eagle’s Medium (DMEM) or Eagle’s Minimum Essential Medium, respectively, and with 10% (*v*/*v*) heat-inactivated fetal bovine serum (FBS, Euroclone, South America origin, EU approved), penicillin (100 U/mL), and streptomycin (100 µg/mL) (Pen/Strep, Euroclone, France). To propagate the viruses, HAdV-2 or HCoV-OC43 liquid stocks (HAdV-2, 1.6 × 10^8^ TCID_50_/mL; HCoV-OC43, 1.6 × 10^6^ TCID_50_/mL) were inoculated respectively onto confluent monolayers of A549 or MRC-5 cells for 90 min in a 2% FBS medium and incubated at 37 °C with 5% CO_2_ until cytopathic effects were observable. Viruses were released from infected cells by three freeze-thaw cycles. The lysates were centrifuged at 2500× *g* for 30 min, and the supernatants were passed through 0.45 then 0.22 µm pore-sized filters (Euroclone, Italy) to remove large debris. The filtrate was purified and concentrated by adding polyethylene glycol 6000 (9% *w*/*v*) and sodium chloride (5.8% *w*/*v*) and stirred overnight at 4 °C. After centrifugation at 10,000× *g* for 45 min, the concentrated viruses were resuspended in 2% FBS DMEM and stored at −80 °C. The virus titer (1.3 × 10^8^ PFU/mL for HAdV-2 and 1.9 × 10^7^ PFU/mL HcoV-OC43) was determined using the agar overlay plaque assay and counting plaques at 4- or 5-days post-infection, respectively. To compare the antibacterial efficacy of the formulations reported in Table 1, MIC_50_ and MIC_100_ were determined using serial dilutions from 100 μL/mL to 6.25 (as reported in Supplementary Figure S1).

### 2.7. Plaque Assay

To evaluate the antiviral effect, different volumes (6.25, 12.5, and 25 µL) of each formulation were incubated simultaneously with the virus at 0.0002 MOI on confluent A549 cells (5 × 10^5^ cells/well, 6-well plate) in 500 μL DMEM supplemented with 2% FBS with periodic shaking for 90 min at 37 °C with 5% CO_2_. At the end of incubation, viral solutions with or without formulations were removed, and infected cells were overlayed with 2.5 mL of a prewarmed agar overlay (DMEM with 2% FBS/Pen/Strep with 0.3% agarose (A5093-500G, Sigma-Aldrich). After 4 days of infection for HAdV-2 and 5 days for HCoV-OC43, infected cells were fixed with 4% (*w*/*v*) paraformaldehyde for 6 h, and after removing the agar overlay, stained with 0.5% (*w*/*v*) crystal violet solution in 20% ethanol, and the number of plaques was counted. Each assay was performed in triplicate on separate days.

### 2.8. Statistical Analyses

The results are expressed as means ± SD. Data were statistically analyzed using an unpaired student *t*-test. A *p*-value of <0.05 was considered statistically significant.

## 3. Results

### 3.1. Antibacterial Activity of the Ophthalmic Formulations

#### 3.1.1. Broth Microdilution Assay

To compare the antibacterial efficacy of the formulations reported in Table 1, MIC_50_ and MIC_100_ were determined using serial dilutions from 100 μL/mL to 6.25 μL/mL (as reported in Supplementary Figure S1).

In Table 2, the MIC_50_ and MIC_100_ of each formulation for *S. aureus* are reported. Dropsept displayed the highest inhibitory effect on *S. aureus*, with an MIC_50_ of 6.25 μL/mL and an MIC_100_ of 9.00 μL/mL (Table 2, Supplementary Figure S1). Two more formulations (Ozodrop and PVA + thymol) showed significant inhibition of *S. aureus* growth, although only when used at higher concentrations (Table 2, Supplementary Figure S1). For *S. epidermidis*, only Dropsept and PVA + thymol completely inhibited bacterial growth at 27.5 μL/mL and 100 μL/mL, respectively, whereas the other solutions did not show any effect (Table 3, Supplementary Figure S1). Regarding Gram-negative bacteria, *E. coli* was fully inhibited only by Dropsept at 30 μL/mL (MIC_100_) and significantly reduced by PVA + thymol with 50 < MIC_50_ < 100 μL/mL (Table 4, Supplementary Figure S1). On the contrary, *P. aeruginosa* growth was only partially inhibited and, in particular, by the thymol and PVA + thymol formulations, which reduced bacterial growth by 30% at 100 µL/mL (Supplementary Figure S1). The remaining solutions had no significant effect on *P. aeruginosa* (Supplementary Figure S1). The latter result is quite impressive given the high virulence of *P. aeruginosa*, as it causes keratitis, is strongly difficult to treat, and has a worse prognosis than other forms of bacterial keratitis [32].

Following the MIC results, we also tested all the formulations to determine the MBC. We found a microbicidal effect only with Dropsept for all bacterial strains, except for *P. aeruginosa*, confirming the MBC values reported by Caruso et al. [33] (Supplementary Figure S2).

#### 3.1.2. Disk Diffusion Assay

**Figure 1:**
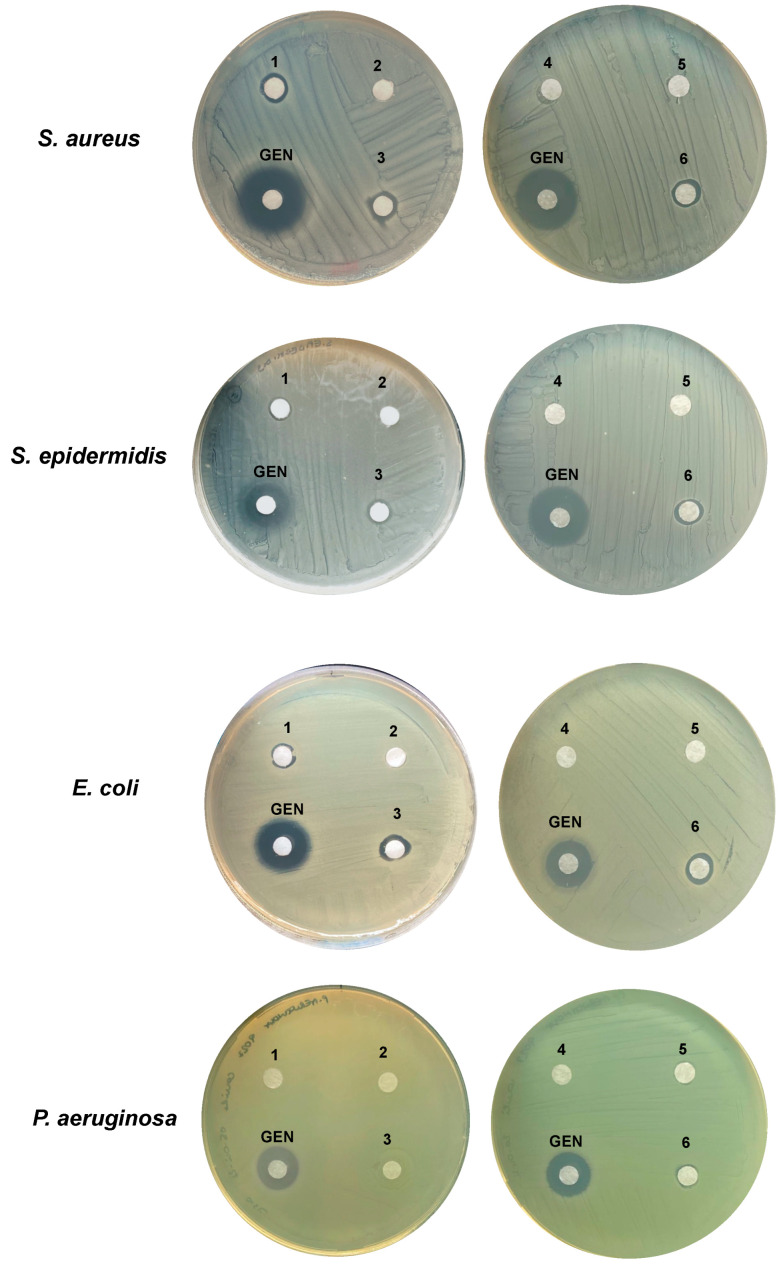
**Disk diffusion assay**. The following ophthalmic formulations were spotted onto agar plates containing the tested microorganism, and the antimicrobial effect was evaluated as inhibition growth zone: 10 μL of Iodim (**1**), Ozodrop (**2**), Dropsept (**3**), PVA (**4**), thymol (**5**), and PVA + thymol (**6**) solutions were assayed respectively for their inhibitory activity over the growth of *S. aureus*, *S. epidermidis*, *E. coli*, and *P. aeruginosa*. Geneticin (GEN) was used as positive control. Representative images of three independent assays were performed in separated days.

In the disk diffusion assay, Iodim, Dropsept, and PVA + thymol solutions inhibited the growth of Gram-positive bacteria with comparable efficiency (Figure 1). On *E. coli*, Dropsept and thymol-based formulations seem to inhibit bacterial growth to a higher extent compared with Iodim, while on *P. aeruginosa*, only PVA + thymol had an inhibitory effect (Figure 1, Table 5). Overall, the results suggest that PVA + thymol formulation exhibits a broad spectrum of antimicrobial activity by the disk diffusion mean.

#### 3.1.3. Challenge Test

The ability of the formulations to prevent microbial growth was tested by the challenge test, in which each formulation was inoculated with *S. aureus*, *S. epidermidis*, *E. coli*, or *P. aeruginosa* cultures, and their growth was monitored over time by counting colonies on agar plates. As shown in Table 6 and Table 7, *S. aureus* and *S. epidermidis* growth was fully inhibited at all the times tested by Iodim, thymol, PVA, and their combination. Dropsept inhibited their growth after 24 h, while Ozodrop totally inhibited the growth of *S. aureus* only after 7 days and of *S. epidermidis* after 24 h (Table 6 and Table 7).

*E. coli* was fully inhibited at each time point by each formulation, except for Ozodrop, which seems to inhibit bacterial growth only after 24 h since the challenge (Table 8).

*P. aeruginosa* growth was fully inhibited by Iodim, PVA, thymol, and their combination at all the time points tested and by Ozodrop only after 7 days (Table 9). Dropsept reduced by five-fold *P. aeruginosa* growth after 2 h of incubation and did not prevent bacteria from recovering at the subsequent time points (Table 9).

### 3.2. Antiparasitic Activity

**Figure 2:**
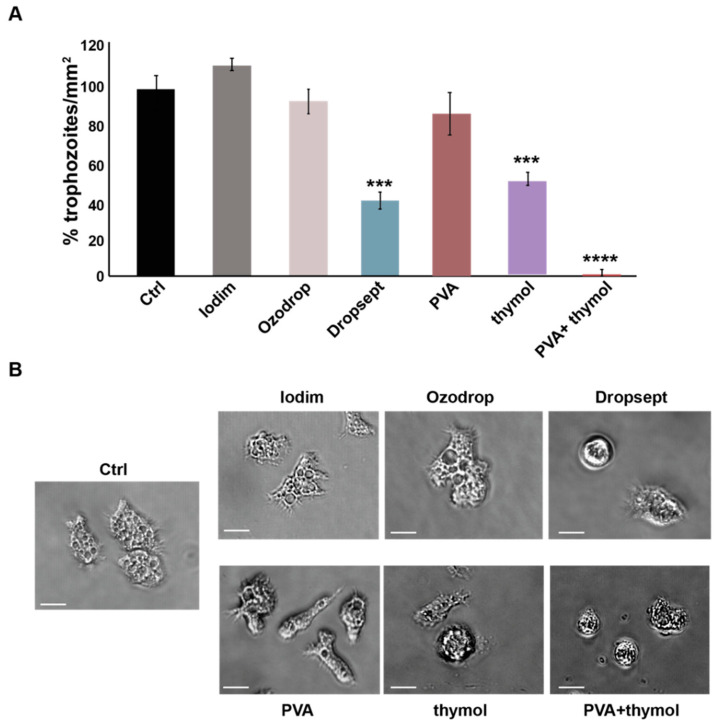
**Antiparasitic activity of the ophthalmic solutions**. (**A**) Histograms of the number of *Acanthamoeba castellanii* trophozoites/mm^2^ exposed to the highest concentrations of each formulation and pictured at 10× magnification. Each formulation was expressed as % of the control (Ctrl, 100%). (**B**) Images of conditions in (**A**) at 40× magnification reveal that the shape of amoeba was particularly compromised in presence of Dropsept and PVA + thymol. Scale bar—10 μm. The results are presented as mean ± SD. *t*-test *** *p* < 0.001, **** *p* < 0.0001 vs. Ctrl.

The antiparasitic activity was assayed by measuring the inhibitory effect of each ophthalmic preparation on *Acanthamoeba castellanii* growth. Briefly, the amoeba was incubated with serial dilutions of each test formulation, and after 48 h, the number of trophozoites was estimated by optical microscopy. In particular, six images per condition were recorded and used to estimate the number of trophozoites. Figure 2A shows the number of trophozoites/mm^2^ exposed to the highest concentrations, 100 μL/mL, of each formulation as a percentage of a control condition. Dropsept and thymol reduced the growth of the amoeba by 50% (MTIC_50_). Moreover, when observed at 40× magnification, the shape of the amoeba incubated with Dropsept was particularly compromised and ultimately dead (Figure 2B). Remarkably, the combination of PVA + thymol completely impaired the formation of trophozoites. These results suggest that Dropsept and thymol-based formulations displayed antiamoebic effects.

### 3.3. Antiviral Activity of the Ophthalmic Formulations

The eye is known to serve as both a potential site of virus replication and an entrance route for spreading the virus to extraocular sites. Given the serious concern about respiratory viral infections, the ophthalmic solutions were also tested for their antiviral properties against two different respiratory viruses, HAdV-2 and HCoV-OC43 [12].

#### 3.3.1. Antiviral Activity against HAdV-2

To determine the in vitro efficacy of ophthalmic formulations against HAdV-2, the virus and each formulation were used simultaneously to infect the A549 cells for 90 min at 37 °C. The viral replication was examined at 4 days post-infection by the plaque formation assay, and the antiviral efficacy was determined by comparing the number of plaques induced by the virus in the presence of the tested formulation *versus* the control condition in which cells were infected only with the virus.

**Figure 3:**
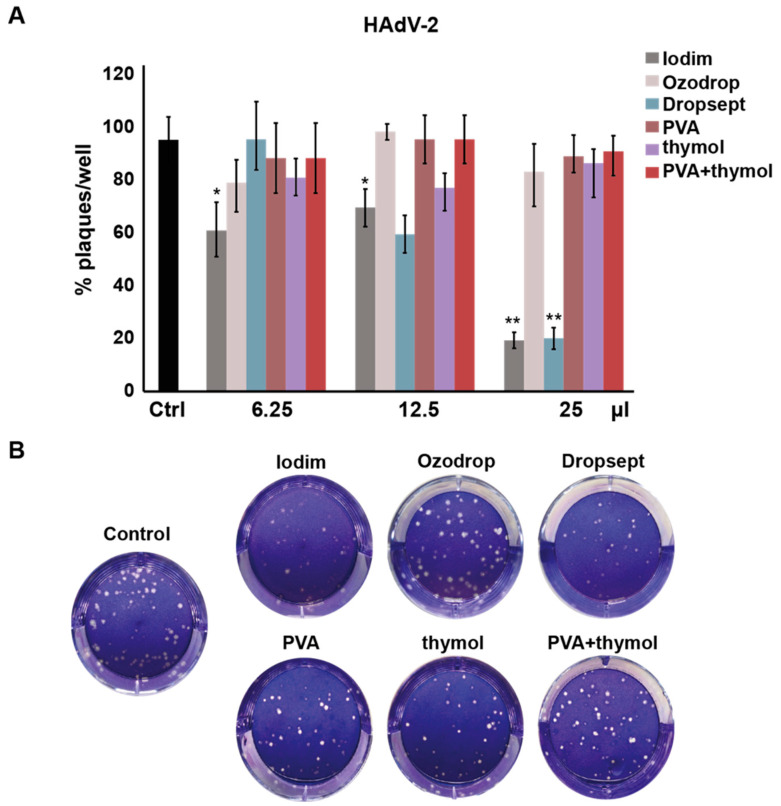
**Antiviral activity of the ophthalmic formulations against HAdV-2**. (**A**) Histograms of the number of plaque-unit forming (PFU) (%) for each test solution at different volumes. The indicated ophthalmic formulations were incubated simultaneously with the virus on A549 cells at different volumes and the plaques monitored after 4 days of incubation. The results are presented as mean ± SD. *t*-test * *p* < 0.05, ** *p* < 0.01 vs. Ctrl. (**B**) Representative pictures of A549 cells incubated with HAdV-2 w/wo formulations (12.5 μL) and stained with crystal violet.

As reported in Figure 3A, Iodim shows considerable inhibition of viral replication with a maximum effect at 25 μL, while Dropsept seems to inhibit the viral replication in a dose-dependent manner. Ozodrop, PVA, and thymol solutions did not exert any antiviral activity against HAdV-2.

#### 3.3.2. Antiviral Activity against HCoV-OC43

To assess the antiviral activity against HCoV-OC43, the formulations were incubated with the virus as described above, and plaque formation was monitored 5 days post-infection. 

**Figure 4:**
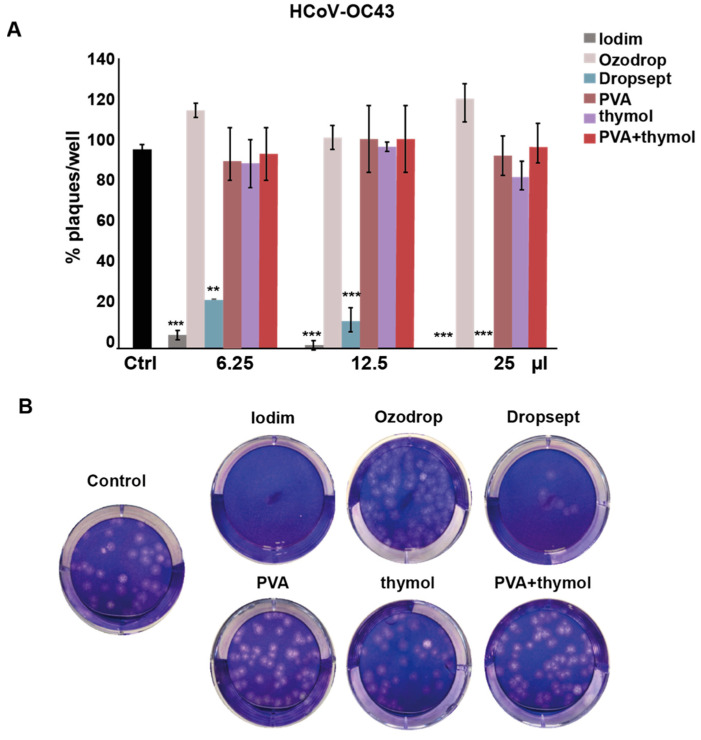
**Antiviral activity of the ophthalmic formulations against HCoV-OC43**. (**A**) Histograms of the number of plaques for each test solution at different volumes. The indicated ophthalmic solutions were incubated simultaneously with the virus on A549 cells at different volumes, and the plaques monitored after 5 days of incubation. The results are presented as mean ± SD. *t*-test ** *p* < 0.01, *** *p* < 0.001 vs. Ctrl. (**B**) Representative pictures of A549 cells incubated with HCoV-OC43 w/wo formulations (12.5 μL) and stained with crystal violet.

As shown in Figure 4A, Iodim and Dropsept produced a dramatic dose-dependent inhibition of viral replication with a maximum effect at 25 μL, whereas Ozodrop, PVA, and thymol solutions did not exhibit any antiviral activity.

Altogether, the results suggest that Iodim and Dropsept have remarkable antiviral properties against the two respiratory viruses tested here.

## 4. Discussion

In the present study, we tested the antibacterial, antiparasitic, and antiviral effectiveness of six ophthalmic formulations, which have the common feature of being constituted by antiseptics rather than antibiotics.

In ophthalmology, the use of antiseptics is becoming increasingly relevant; indeed, topical antiseptics could be valuable alternatives to antibiotics since they present nonselective mechanisms of action preventing bacterial resistance [16,18]. Their effectiveness can be exploited for treating either newly developed ocular infections or as prophylaxis in pre- and post-surgery infections. It is frequent that patients following ocular surgery might suffer from endophthalmitis in the postoperative period due to bacteria residing in the conjunctiva. Moreover, the antibiotic-based strategy to prevent this kind of ocular infection is now proven to induce antibiotic resistance [34,35]. For these reasons, antiseptics are now becoming a more proper therapeutic approach in ophthalmology.

The bacterial strains enrolled in this work were chosen because of their tight correlation with different types of ocular infections, such as blepharitis, conjunctivitis, and keratitis [36]. Therefore, we evaluated the antibacterial activity of each formulation against *S. aureus* and *S. epidermidis* (Gram-positive bacteria) and *E. coli* and *P. aeruginosa* (Gram-negative bacteria). Several chronic ocular infections are also caused by protozoan parasites that form cysts within the tissue; thus, to assess the antiparasitic potential of each ophthalmic solution, we compared the MTIC_50_ of each formulation against *A. castellanii*, an opportunistic pathogen that is associated with blinding eye keratitis [37]. Ultimately, as the eye might also be infected by respiratory viruses causing human ocular diseases and giving entry to the extraocular sites, we evaluated the antiviral activities of the formulations against two respiratory viruses.

Among all the ophthalmic formulations here assayed, the results of our study highlight Dropsept as the formulation with the highest efficiency, as it showed a broad spectrum of action against the tested bacteria, the amoeba, and the two respiratory viruses. Therefore, Dropsept, because of its elevated safety, as shown in Caruso C. et al. [38], and its effectiveness might represent a promising therapy before ophthalmic and intravitreal surgery in preventing post-surgery endophthalmitis. In addition, its inhibitory effect against HAdV-2 and HCoV-OC43 also makes this formulation suitable for preventing or limiting viral infections.

The overall results show that Dropsept exerts the highest inhibitory effect against bacteria. Indeed, it was the only formulation able to fully inhibit bacterial growth and elicit bactericidal action on each bacterial strain, except for *P. aeruginosa*. However, MIC_50_ values on *S. aureus* were also detected for other ophthalmic solutions such as Ozodrop and PVA + thymol, revealing that among the tested bacteria, *S. aureus* was the most affected by the different formulations. A significant inhibitory effect on *S. epidermidis* and *E. coli* was also observed with PVA + thymol. Even if Dropsept was the most effective ophthalmic solution, no effect was observed on *P. aeruginosa*, whereas the PVA + thymol solution showed a significant inhibitory effect on this bacterial strain. Given the increasing concern about the antibiotic resistance of *P. aeruginosa*, which developed over the years several strategies to counteract the effect of antibiotics, including biofilm formation and multidrug-tolerant persister cells, any alternative therapeutic approach able to limit the recalcitrance is now urgently required [39]. Here, we observed an inhibition zone in the disk diffusion assay and a reduction of *P. aeruginosa* growth up to 30% in the presence of PVA + thymol, which represents a remarkable result and might provide a promising therapeutic approach for treating *P. aeruginosa*-caused infections (Figure 1, Supplementary Figure S1).

The preservative properties of these formulations were also tested by a challenge test for up to 7 days. According to the results, each formulation prevents bacterial growth once challenged, with the exception of Dropsept, which was not able to limit *P. aeruginosa.* Our data are not in line with Tognetto et al. (2022), who published a microbicidal effect only for Iodim [40].

Regarding the antiparasitic activity against *Acanthamoeba castellanii*, Dropsept was the most effective solution, along with thymol-based solutions, as they reduced the proliferation rate of the amoeba remarkably. The effect observed with Dropsept was in line with in vivo data published by Caruso C. et al., which showed a favorable clinical outcome in patients affected by *Acanthamoeba keratitis* and treated with Dropsept [38].

Ultimately, Dropsept and Iodim exhibited a significant dose-dependent antiviral activity against HAdV-2 and HCoV-OC43. In particular, the replication of human betacoronavirus was fully inhibited by 25 μL of these two formulations, whereas unaffected by the other formulations.

Based on the described data, Dropsept emerged as the most effective formulation capable of eliciting multiple activities, antibacterial, antiparasitic, and antiviral ones. The only limitation of this formulation regards *P. aeruginosa*, which did not respond to its application. The refractory response to Dropsept might be due to a progressive low membrane permeation to CHX, along with an adaptation of the bacterial cell membrane resulting in changes in intracellular biochemical processes [41,42]. Exposure to subliminal concentrations of CHX could reduce the risk of cell adaptation in *P. aeruginosa* [43]. The higher susceptibility of Gram-positive rather than Gram-negative bacteria to the tested ophthalmic solutions, particularly to Dropsept, is in line with the literature, which considers the outer membrane of Gram-negative bacteria as the major barrier against antibiotics or antimicrobials [44].

Dropsept was also greatly promising in affecting *A. castellanii* compared with the other solutions. In this context, CHX, contained in Dropsept, often in association with polyhexamethylene biguanide (PHMB), is the most effective topical treatment against *Acanthamoeba*, working against both amoebic forms (cysts and trophozoites) [45]. The parasite-killing effect of CHX resides in its highly charged positive molecules that penetrate the amoeba and binds the phospholipid bilayer of the cell membrane, negatively charged, producing cell lysis and death [46]. Thus, it was not surprising that the combination of Vitamin E TPGS CHX increased the effect of CHX inhibiting *A. castellanii* growth significantly and affecting its shape. Similar to Dropsept, thymol, particularly in combination with PVA, was able to significantly reduce the degree of growth of *A. castellanii*, in line with a report about the antiparasitic effect of thymol-based essential oils from *Ammoides pusilla* [47]. Because of its efficient antiparasitic and antimicrobial activity, particularly against *P. aeruginosa*, thymol might also represent another promising strategy for treating a variety of ocular infections. In addition to the antiamoebic effect, PVA + thymol also showed an effect against the tested bacterial strains with MIC_50_ values of 50 μL, 50 μL, and 50 < MIC_50_ < 100 μL, respectively, on *S. aureus*, *S. epidermidis*, and *E. coli* and a 30% reduction in *P. aeruginosa* when used at their maximal concentration. It is not surprising that thymol exhibited such an antimicrobial effect, as it is reported to interact with the lipid bilayer of cytoplasmatic membranes causing loss of integrity and leakage of cellular material [48]. In particular, it is remarkable the effect on *P. aeruginosa*, which has developed different mechanisms for surviving antiseptics and antibiotics [47]; from this perspective, thymol could represent a novel alternative therapeutic approach to the conventional antibiotic-based treatment.

Several pieces of evidence report the efficacy of antiseptic solutions to inhibit viral replication in vitro, and viral inactivation seems to occur by different mechanisms, for instance, chemically modifying viral surface groups or dissolution of lipid envelop [49]. The virucidal activity is variable according to the antiseptic molecules; for instance, povidone-iodine is reported to be more effective than chlorhexidine on nonenveloped viruses, although their efficacy depends greatly on the composition of each formulation. Here, we found that two different antiseptic solutions, Iodim and Dropsept, show a remarkable virucidal effect on two viruses, enveloped and nonenveloped, HAdV-2 and HCoV-OC43, both responsible for respiratory infections. 

## 5. Conclusions

Overall, the main finding of the present work shows that among the tested ophthalmic solutions, the commercial eye drop solution Dropsept, containing Vitamin E TPGS and CHX [28], is active against bacteria (*S. aureus*, *S. epidermidis*, and *E. coli*), *Acanthamoeba castellanii* and two respiratory viruses (HAdV-2 and HCoV-OC43), commonly associated with ocular infections. Particularly, Dropsept is a recently developed formulation able to overcome the limits of CHX’s use and showing high penetration through the human cornea [33]. Given its multiple activities, Dropsept might be enrolled as a valuable alternative to treat ocular infections compared with topical liniments and antibiotics gels or to prevent post-surgical endophthalmitis. Promising results were also shown by thymol, which had inhibitory effects, particularly against *P. aeruginosa*, frequently responsible for resistant ocular infections, and against *A. castellanii*, known to cause severe keratitis. Finally, thymol might also represent another possible treatment for ocular infections that will not trigger drug resistance.

## Figures and Tables

**Table 1:** List of the ophthalmic solutions.

Ophthalmic Solutions	Formulation
Iodim (Medivis s.r.l, Catania, Italy)	0.6% PVP-I, medium-chain triglycerides (MCTs), sodium hyaluronate and glycerol
Ozodrop (FB Vision s.r.l, San Benedetto del Tronto (AP) Italy)	Lipozoneye (ozonated sunflower oil, soy phospholipids), hydroxypropylmethylcellulose, polyhexamethylene biguanide (PHMB), boric acid, sodium tetraborate, sodium edetate, disodium, and deionized water
Dropsept (Iromed Group s.r.l, Roma, Italy)	D-alpha-tocopherol poly (ethylene glycol) 1000 succinate (Vitamin E TPGS) (0.2%) and CHX digluconate solution (0.02%)
PVA	0.75% polyvinyl alcohol
Thymol	0.5% thymol
PVA + thymol	0.75% polyvinyl alcohol + 0.5% thymol

**Table 2:** *S. aureus* MIC_50_ and MIC_100_.

Ophthalmic Formulations	MIC_50_	MIC_100_
Iodim	>100	>100
Ozodrop	100	>100
Dropsept	6.25	9.00
PVA	>100	>100
Thymol	>100	>100
PVA + thymol	50	100

**Table 3:** *S. epidermidis* MIC_50_ and MIC_100_.

Ophthalmic Formulations	MIC_50_	MIC_100_
Iodim	>100	>100
Ozodrop	>100	>100
Dropsept	18.0	27.5
PVA	>100	>100
Thymol	>100	>100
PVA + thymol	50	100

**Table 4:** *E. coli* MIC_50_ and MIC_100_.

Ophthalmic Formulations	MIC_50_	MIC_100_
Iodim	>100	>100
Ozodrop	>100	>100
Dropsept	20	30
PVA	>100	>100
Thymol	>100	>100
PVA + thymol	50 < MIC_50_ < 100	>100

MIC_50_ and MIC_100_ were expressed as µL/mL.

**Table 5:** Schematic results of disk diffusion assay.

Formulations	*S. aureus*	*S. epidermidis*	*E. coli*	*P. aeruginosa*
1	+	+	+	−
2	−	−	−	−
3	+	+	+	−
4	−	−	−	−
5	−	−	−	-
6	+	+	+	+
GEN	+++	+++	+++	+++

Symbols + or − refer respectively to the presence or absence of bacterial growth inhibition.

**Table 6:** Challenge test on *S. aureus*.

Formulations	T 2 h	T 24 h	T 7 d
	Log Reduction		
Iodim	NR ^#^	NR	NR
Ozodrop	R ^^^	>1	NR
Dropsept	R	NR	NR
PVA	NR	NR	NR
Thymol	NR	NR	NR
PVA + thymol	NR	NR	NR

^#^ Not recover; ^^^ recover.

**Table 7:** Challenge test on *S. epidermidis*.

Formulations	T 2 h	T 24 h	T 7 d
	Log Reduction		
Iodim	NR	NR	NR
Ozodrop	R	NR	NR
Dropsept	>3	NR	NR
PVA	NR	NR	NR
Thymol	NR	NR	NR
PVA + thymol	NR	NR	NR

**Table 8:** Challenge test on *E. coli*.

Formulations	T 2 h	T 24 h	T 7 d
	Log Reduction		
Iodim	NR	NR	NR
Ozodrop	R	NR	NR
Dropsept	NR	NR	NR
PVA	NR	NR	NR
Thymol	NR	NR	NR
PVA + thymol	NR	NR	NR

**Table 9:** Challenge test on *P. aeruginosa*.

Formulations	T 2 h	T 24 h	T 7 d
	Log Reduction		
Iodim	NR	NR	NR
Ozodrop	R	>2	NR
Dropsept	>5	R	NI ”
PVA	NR	NR	NR
thymol	NR	NR	NR
PVA + thymol	NR	NR	NR

” Not induction.

## Supplementary Materials

The following are available online at https://www.mdpi.com/article/10.3390/microorganisms10061156/s1, Figure S1: Minimum Inhibitory Concentration (MIC) of ophthalmic formulations, Figure S2: Minimum bactericidal concentrations (MBC) of ophthalmic formulations.
