# Supplementary material for: Comparative Evaluation of Antimicrobial, Antiamoebic, and Antiviral Efficacy of Ophthalmic Formulations
Source: Microorganisms. 2022 Jun 4;10(6):1156. doi: 10.3390/microorganisms10061156 (PMC9229167; doi:10.3390/microorganisms10061156)
Supplement: Supplementary file 1 [file microorganisms-10-01156-s001.zip › microorganisms-1746161-supplementary.pdf]

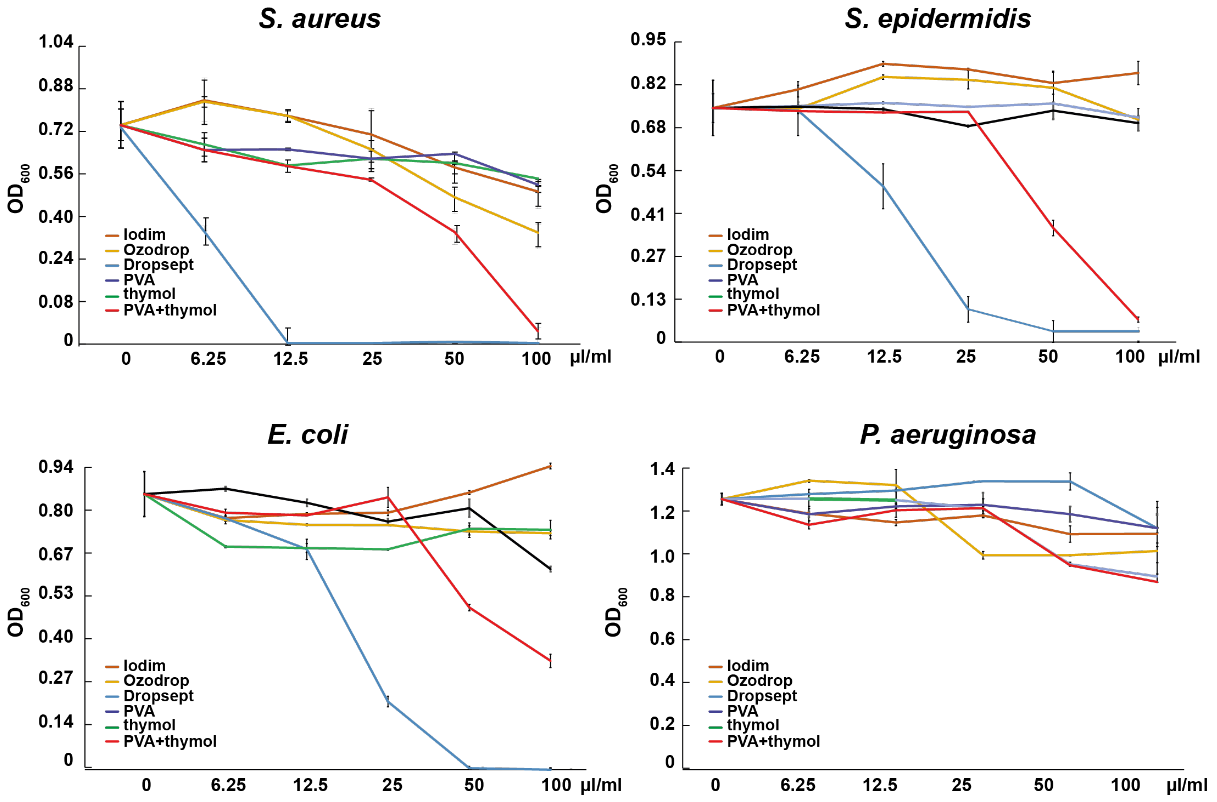

Figure S1. Minimum Inhibitory Concentration (MIC) of ophthalmic formulations. *S. aureus, S. epidermidis*. *E. coli* and *P. aeruginosa* were grown for 24 hours in presence of two-fold serial dilutions of the indicated formulations and measured at 600nm. The results are expressed as mean +/- SD. Note that these solutions were tested up to 100 µL/mL.

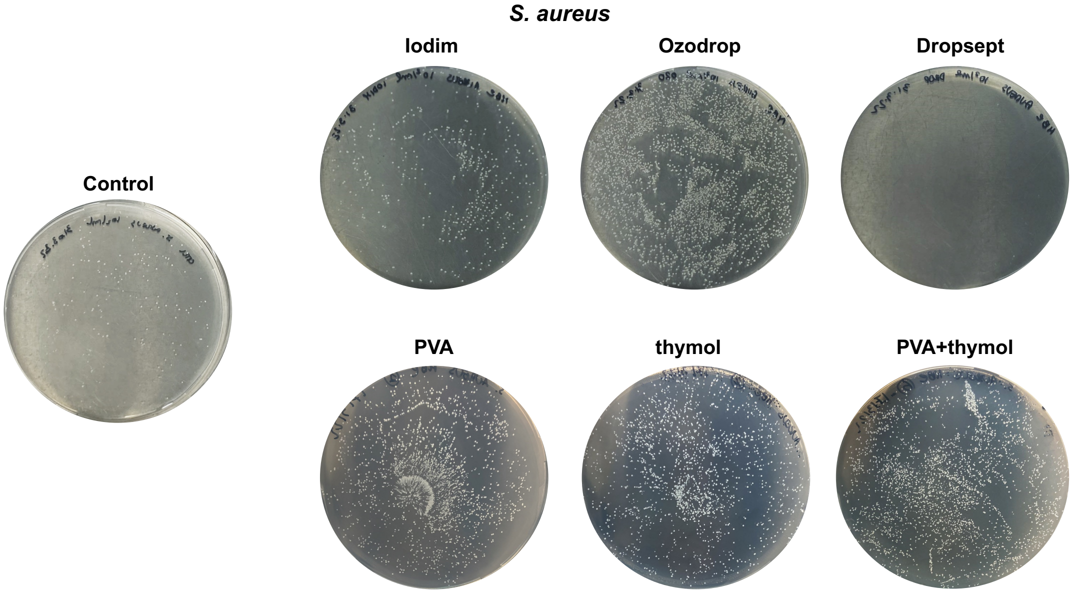
S. aureus
Iodim
Ozodrop
Dropsept
Control
PVA
thymol
PVA+thymol

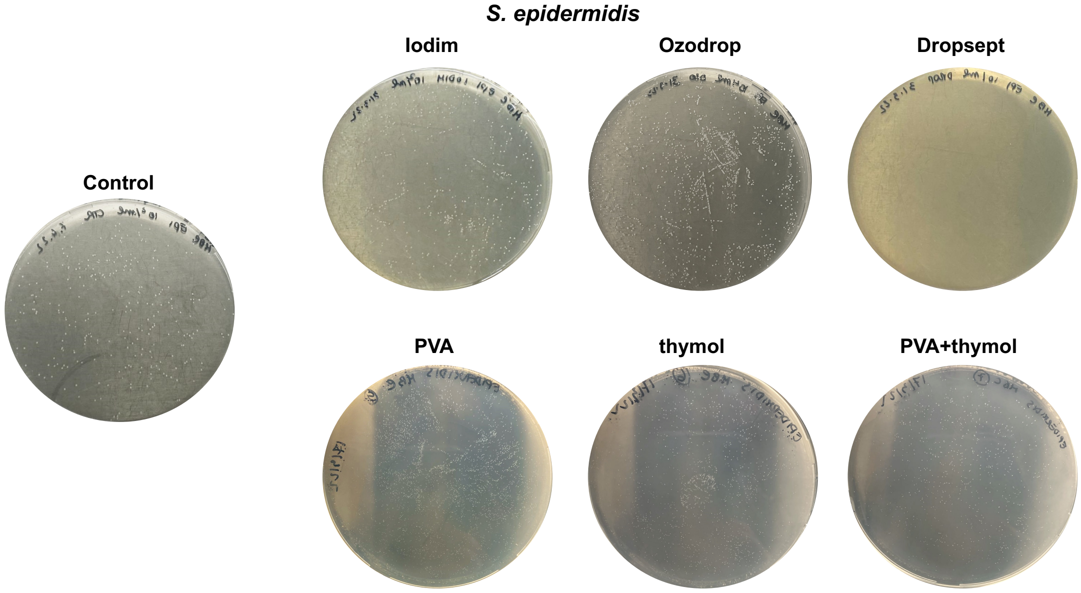
S. epidermidis
Iodim
Ozodrop
Dropsept
Control
PVA
thymol
PVA+thymol

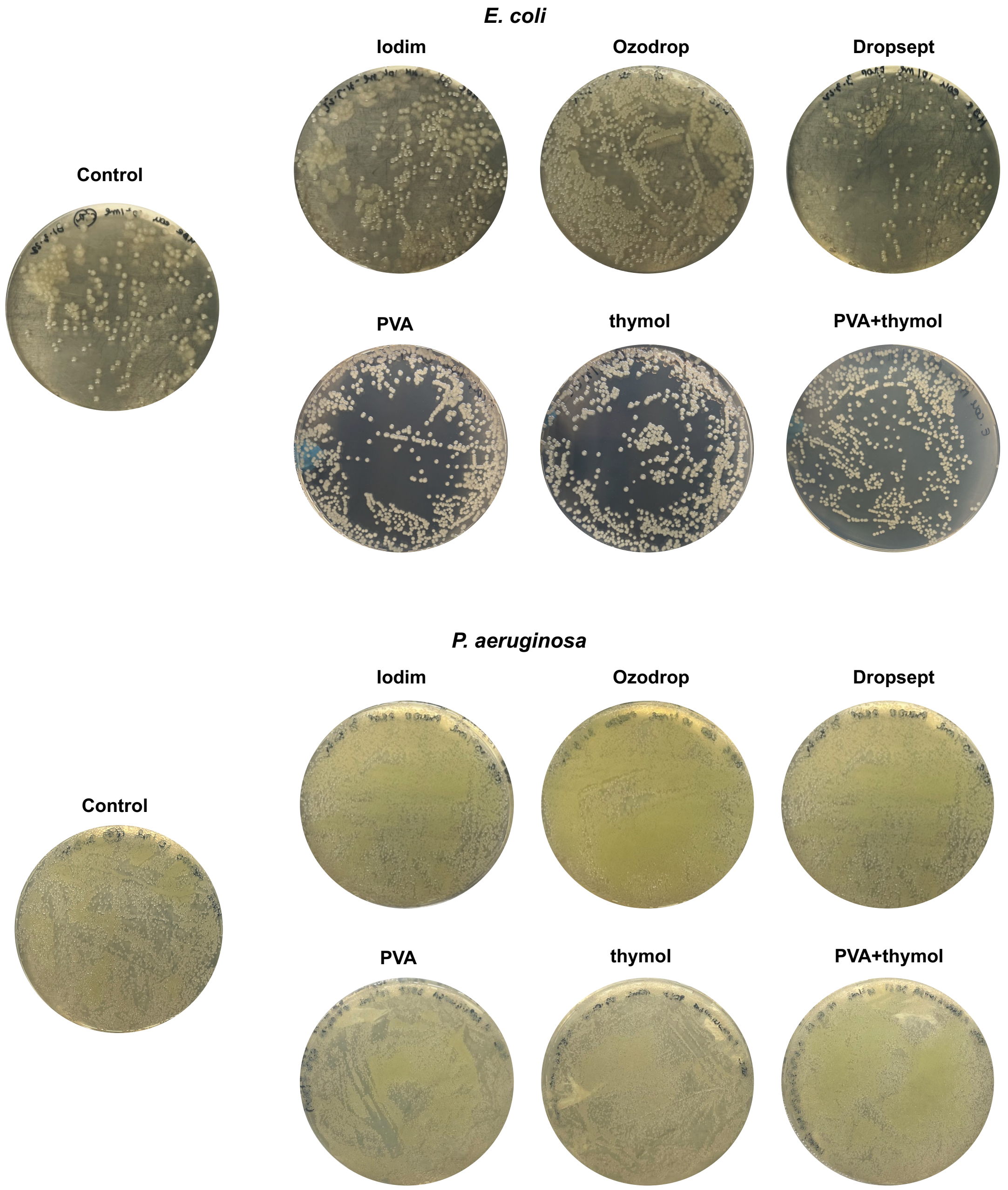

Figure S2. Minimum bactericidal concentrations (MBC) of ophthalmic formulations. *S. aureus, S. epidermidis*. *E. coli* and *P. aeruginosa* were grown for 24 hours in presence of 100 µL/mL of the indicated formulations and the bactericidal effect was determined by plating proper dilutions on MHA plates. The images are representative of experiments performed in triplicate.
